# Ultrasound of Bile Ducts—An Update on Measurements, Reference Values, and Their Influencing Factors

**DOI:** 10.3390/diagnostics15070919

**Published:** 2025-04-02

**Authors:** Claudia Lucius, Anja Flückiger, Jennifer Meier, Kathleen Möller, Christian Jenssen, Barbara Braden, Michael Kallenbach, Benjamin Misselwitz, Christian Nolsøe, Michael Sienz, Constantinos Zervides, Christoph Frank Dietrich

**Affiliations:** 1 Outpatient Department of Gastroenterology, IBD Centre Helios Hospital Berlin Buch, 13125 Berlin, Germany; claudia.lucius@gmx.de; 2 Department General Internal Medicine (DAIM), Hospitals Hirslanden Bern Beau Site, Salem and Permanence, 3013 Bern, Switzerland; anja.flu@hotmail.com; 3 Department Allgemeine Innere Medizin, Kliniken Hirslanden, Beau Site, Salem und Permanence, 3013 Bern, Switzerland; jenny.meier-grau@hotmail.ch; 4 Medical Department I/Gastroenterology, SANA Hospital Lichtenberg, 10365 Berlin, Germany; 5 Department for Internal Medicine, Krankenhaus Märkisch Oderland, 15344 Strausberg, Germany; 6 Brandenburg Institute for Clinical Ultrasound (BICUS) at Brandenburg Medical University, 16816 Neuruppin, Germany; 7 Translational Gastroenterology Unit, Oxford University Hospitals, Oxford OX3 9DU, UK; barbara.braden@ukmuenster.de; 8 Medical Department B, University Muenster, 48149 Muenster, Germany; 9 Klinik für Gastroenterologie, Hepatologie und Infektiologie, Universitätsklinikum Düsseldorf, 40225 Dusseldorf, Germany; 10 Department of Visceral Surgery and Medicine, Bern University Hospital, University of Bern, 3012 Bern, Switzerland; 11 Medizinische Klinik und Poliklinik II, LMU München, 80539 München, Germany; 12 Center for Surgical Ultrasound, Department of Surgery, Zealand University Hospital, 4600 Køge, Denmark; 13 Institute for Clinical Medicine, Denmark University of Copenhagen, 1172 Kobenhavn, Denmark; 14 Benedictine Congregation of St. Ottilien, St. Benedict Hospital Ndanda, Ndanda, Mtwara Region, Tanzania; 15 CZMH Medical Physics and Dosimetry Services Ltd., Limassol, Cyprus; c.zervides@czmh.org

**Keywords:** reference values, common bile duct, diameters, measurement, cystic duct, bile duct drainage

## Abstract

**Objective:** To provide an overview of the technique and normal values of ultrasound studies of the bile system based on the published literature. **Methods:** A literature search for ultrasound studies with measurements of the bile ducts in healthy subjects was performed. Relevant data published between 1975 and end of 2024 were extracted, discussed, and complemented with the own experiences of the authors. The clinical implications are presented and discussed. **Results:** For the diameter of the common bile duct, reference values between 5 and 9 mm have been published. The main influencing factors are age and history of cholecystectomy, and other factors to be considered are discussed here. The cut-off for the common bile duct wall is set at 1.5 mm. The literature on measurements of intrahepatic bile ducts is scarce. A diameter of <2–3 mm can be considered normal. The method of ultrasound examination is presented here, as well as a comparison with other imaging methods and their clinical implications. **Conclusions:** Standardized measurement techniques and normal values in the context of influencing factors are crucial for the ultrasound examination of the bile system.

## 1. Introduction

Ultrasound examination of the liver and bile system is usually the first-choice imaging modality for patients presenting with abdominal problems [1,2]. Normal values for measurements of the bile ducts are crucial for the discrimination of pathological changes, as well as for the decision management of patients. The literature on reference values for bile duct measurements is controversial due to different study populations and measurement techniques [3].

In this review, we provide a comprehensive survey of the literature on the techniques and measurements of ultrasound scans of the bile system and discuss their clinical impact.

## 2. Review of the Literature

The literature on reference values in abdominal ultrasound was reviewed based on three German-language publications published between 2010 and 2012 by Sienz et al. [3,4,5], which will not be repeated, but rather complemented with the literature published from 2010 until December 2023. In a series of papers, the use of measurements and the knowledge of reference values regarding other organ systems are being updated [6,7,8,9,10].

### 2.1. Search Strategy

PubMed was the database searched for entries from 1 January 1975 until 3 January 2025 using the following: (bile duct OR common bile duct OR extrahepatic bile duct) AND (ultrasound [title/abstract] OR ultrasonography [title/abstract] OR sonography [title/abstract] OR sonographic* [title/abstract]) AND (measurement OR sizing OR diameter OR width OR height OR length OR “reference value” OR “normative value” OR “cut-off value”). From this method, 1220 entries were identified in PubMed (final search date: 3 January 2025).

### 2.2. Study Selection

Two of the authors independently reviewed the titles and abstracts for eligibility. Animal studies, studies related only to pediatric cohorts (0–14 years), case studies (<10 cases), editorials, letters to the editors, full-text articles without English, German, French, or Spanish text, duplicates, articles not referring to the bile duct, articles including only measurements of pathologic conditions of the bile duct, and articles only including non-ultrasound imaging modalities were excluded. Articles already included in Sienz et al.’s reference list were evaluated separately. They were included partially, as the review was published in the German language and not all clinical implications were discussed [3]. Extensive cross-checking of the reference list of the retrieved articles was also performed. Disagreements regarding eligibility were resolved by discussion and consensus among all authors.

### 2.3. Data Extraction

**Figure 1:**
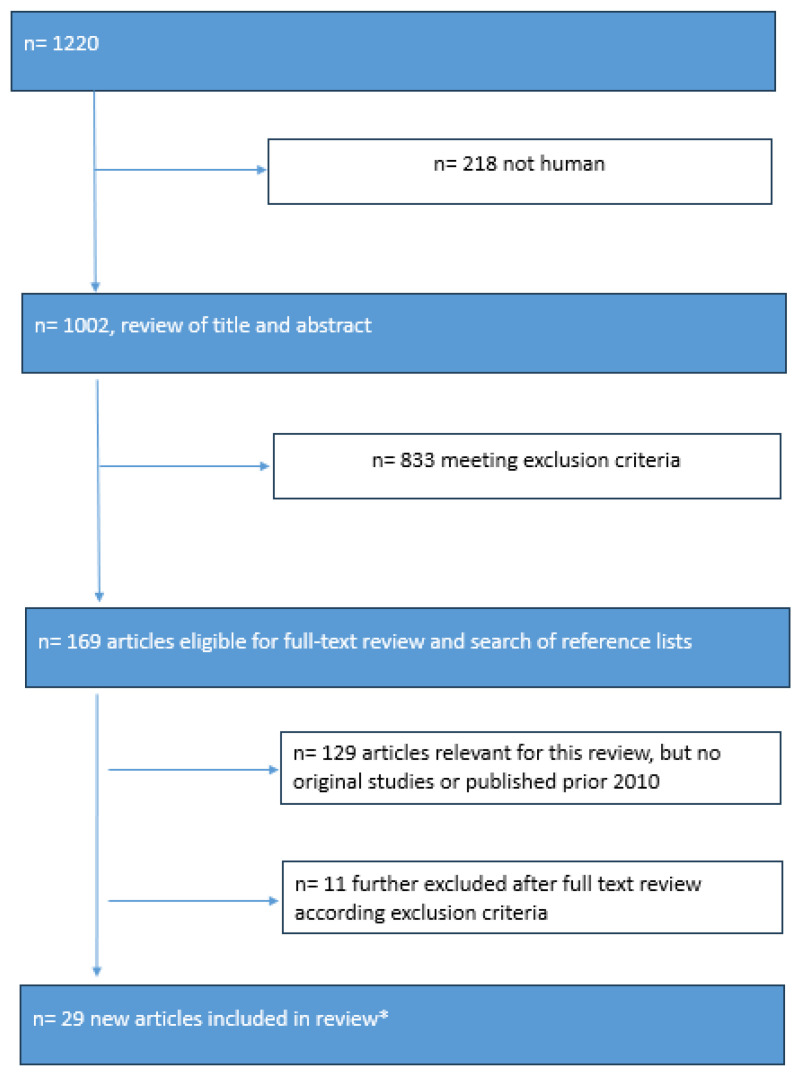
Results of literature search summarized in flow chart. Flow chart describing search strategy and selection of studies finally included in this review. * Further references with important content were included from 2010 and earlier, when not evaluated in the review by Sienz et al. or concerning additional clinical settings.

The data were extracted based on the year of publication and imaging method used for assessment (e.g., transcutaneous ultrasound, endoscopic ultrasound, computed tomography (CT), magnetic resonance cholangiopancreaticography (MRCP), endoscopic retrograde cholangiopancreaticography (ERCP), bile duct wall, bile duct diameter, and bariatric surgery). For the search results, see the flow chart [Figure 1].

### 2.4. Summary of the Literature Published Before 2010

The width of the common bile duct (CBD) is an important diagnostic parameter for distinguishing obstructive from non-obstructive jaundice [11]. The normal values were defined between 5 and 9 mm in older patients depending on the location of measurement and on the study population [12]. There is no consensus on the upper range of the common bile duct diameter after cholecystectomy [13,14,15,16,17,18]. Diameters of <3 mm for the right and left hepatic ducts were measured by Dewbury et al. in 1980 [19]. The hepatic ducts were considered normal when measuring half of the associated portal vein branches. A sharp ending of the bile duct might hint at malignancy [20]. Clinical studies on the visualization and normal values of the cystic duct do not yet exist. Yarmenitis described the cystic duct as a tortuous tubular structure of about 2–3 cm in length, often causing a sonic shadow [1], which, in most cases, can be visualized in a slight left-sided position [3].

### 2.5. CBD Diameter—Summary of the More Recently Published Literature Since 2010

In a single-center study conducted by Matcuk et al. [21], they retrospectively analyzed 4119 ultrasound examinations of the extrahepatic duct (EHD), right intrahepatic duct (RIHD), and left intrahepatic duct (LIHD). The EHD was measured longitudinally anterior to the portal vein, near the intersection of the right hepatic artery. The intrahepatic ducts were assessed at the level of the portal bifurcation. The mean normal values for EHD, RIHD, and LIHD were 3.8 ± 1.6 mm, 1.9 ± 1.9 mm, and 1.9 ± 1.1 mm.

In a descriptive study conducted by Ongoiba et al. [22], normal values of the origin and the end of the CBD were analyzed in 60 healthy subjects (31 males, 29 females; age 20–39 years) using a 3.5 MHz curved probe and a 7.5 MHz linear probe. The mean diameter was 2.6 ± 1.4 mm at the origin and 3.1 ± 0.7 mm at the end. The distal measurements were significantly more reliable compared to the proximal measurements.

In a retrospective data analysis of 8.534 patients (4.054 male, 4.480 female; age 59.2 ± 18 years), Kratzer et al. [23] investigated the influence of different factors on CBD caliber in patients with and without a history of cholecystectomy. A 2–5 MHz transducer was used. With a fan-like motion, the liver parenchyma and intrahepatic bile ducts were analyzed in sub- and/or intercostal planes. The extrahepatic bile duct was identified at the midportion level of the portal vein, with the hepatic artery crossing perpendicularly between them or, if not possible, at the widest inner diameter of the extrahepatic bile duct. Overall values of 5.3 ± 3.0 mm were measured, with no statistically significant difference regarding CBD diameter with or without a history of cholecystectomy (5.7 ± 3.3 mm vs. 5.2 ± 2.9 mm).

A cut-off value of 7 mm for normal EHD was confirmed in several studies. The influence of cholecystectomy on CBD diameter was investigated in further studies. A retrospective study by McArthur et al. analyzing 893 patients over a period of six years with two sonographic CBD assessments in three groups (1 = cholecystectomy before first US, 2 = cholecystectomy between first and second examination, 3 = no cholecystectomy) confirmed the hypothesis of CBD dilation after cholecystectomy [24]. In the group of subjects who never had gallstones or cholecystectomy, the mean CBD diameters were 3.44 ± 1.15 mm and 3.77 ± 1.21 mm in the first and second assessments, respectively, showing a slight age-dependent increase.

A longitudinal Croatian study by Valkovic et al. assessing CBDs before and after cholecystectomy found a slight increase in CBD diameter 3 and 6 months postoperatively, but still below the pathological threshold [25]. They evaluated three different points for measurements. The overall preoperative mean diameter of CBDs was 3.32 ± 0.99 mm, which was comparable to the control group of 50 healthy subjects.

Radmard et al. investigated the influence of opium consumption on CBD diameter in an Iranian population. Within the control group of 1432 subjects, the CBD diameter was 4.74 ± 1.34 mm [26].

Furthermore, there are several new studies focusing on comparisons with other imaging methods or the influence of different clinical settings. They will be discussed below in separate sections within the specific clinical context.

Altogether, the use of the anatomical terms in literature varies a lot, sometimes within one study. Therefore, we decided to substitute Common Bile Duct (CBD) by Extrahepatic Bile Duct (EHD) according to the generally accepted anatomic definition. Here the extrahepatic bile duct is defined anatomically as follows: the section close to the liver = “common hepatic duct (CHD)” extends from the bifurcation of the left and right intrahepatic bile duct to the insertion of the Dc. cysticus. The union of CHD with the cystic duct is referred to as the Dc. choledochus or common bile duct (CBD).

## 3. Indications

The indications for ultrasound examination of the bile ducts are as follows, among others: differential diagnosis of jaundice, right upper abdominal pain, elevated liver function tests, and known or suspected tumor of the pancreas or biliary system.

## 4. Examination Technique

### 4.1. Prerequisites for Measurement (e.g., Transducer Type and Frequency, Position of the Patient)

#### 4.1.1. Patient Preparation

- None.

#### 4.1.2. Patient Position (Compare Figure 2a–f)

- Supine position (standard option).
- 15–30° left lateral oblique position (best option).
- Seated or standing position.

**Figure 2:**
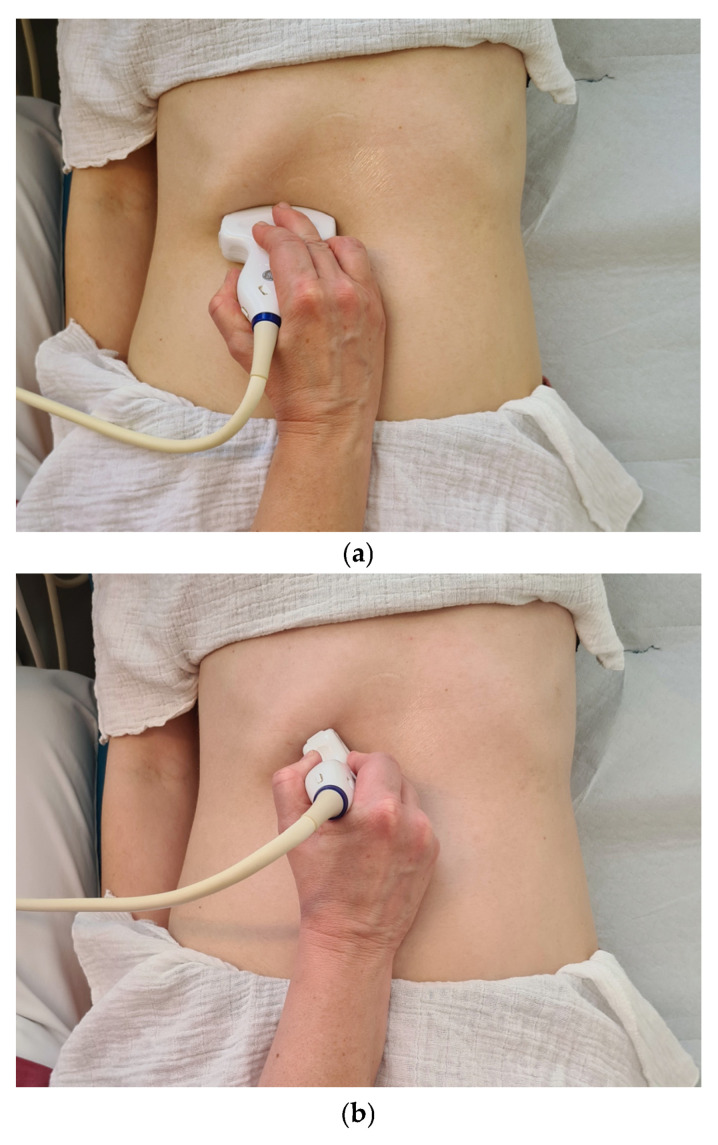

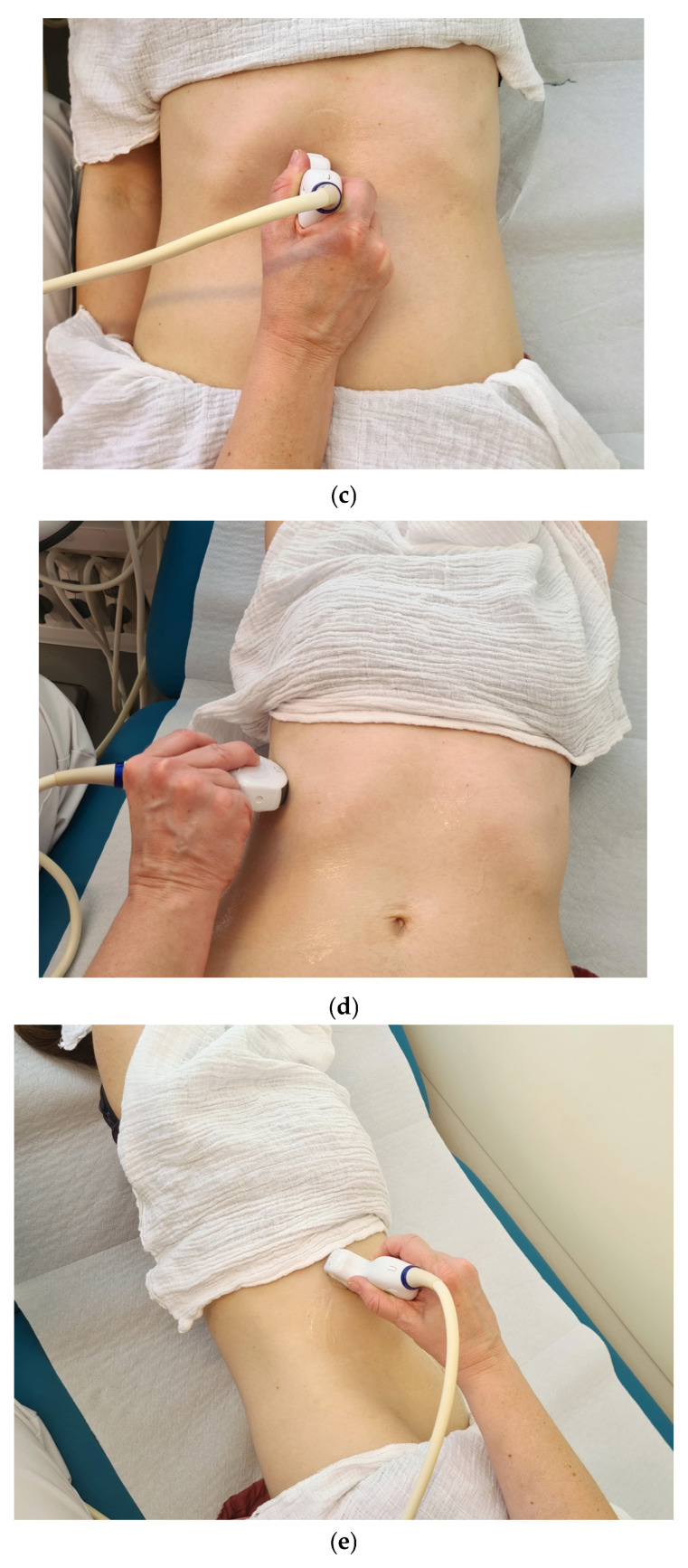

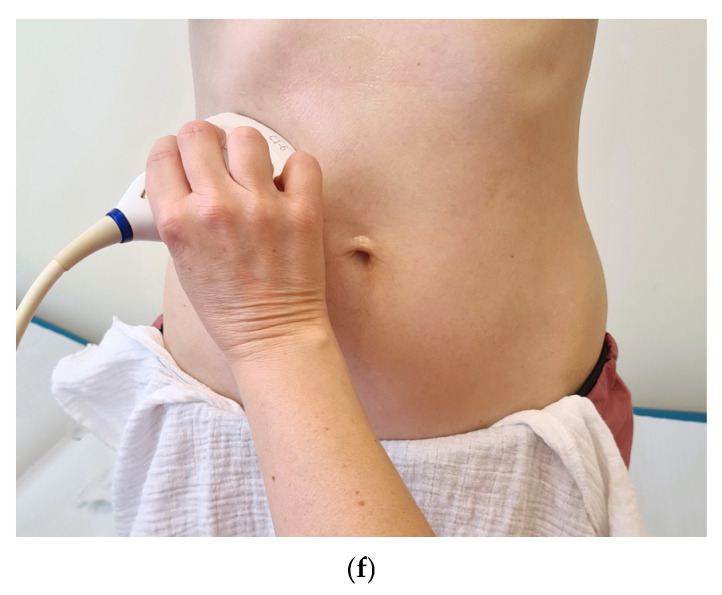
Possible patient positions and corresponding transducer positions for (**a**) supine position with subcostal, more horizontal scan of proximal bile ducts with confluence of common hepatic ducts (CHD). (**b**) Supine position with subcostal shoulder–navel section for liver hilus with CHD and CBD. (**c**) Supine position with longitudinal scan of distal retropancreatic CBD. (**d**) Supine position with intercostal view of CHD and hilus. (**e**) Left lateral oblique position with subcostal shoulder–navel scan. (**f**) Standing position. For all maneuvers, deep inhalation and a certain amount of compression with the transducer can be helpful.

#### 4.1.3. Transducer Type and Initial Position (Compare Figure 2a–f)

- Standard abdomen 2–7 MHz multifrequency curvilinear probe, positioned subcostally in the shoulder–navel orientation.

The examination should be performed with the transducer in a sagittal subcostal position and right oblique orientation (shoulder–navel section) in order to first localize the common hepatic duct (CHD) in the liver hilus [23]. To follow the course of the common bile duct (CBD) along its longitudinal axis, the transducer is rotated clockwise step by step and moved medially and caudally at the same time. The transducer will then be aligned sagittally at the level of the pancreatic head.

**Figure 3:**
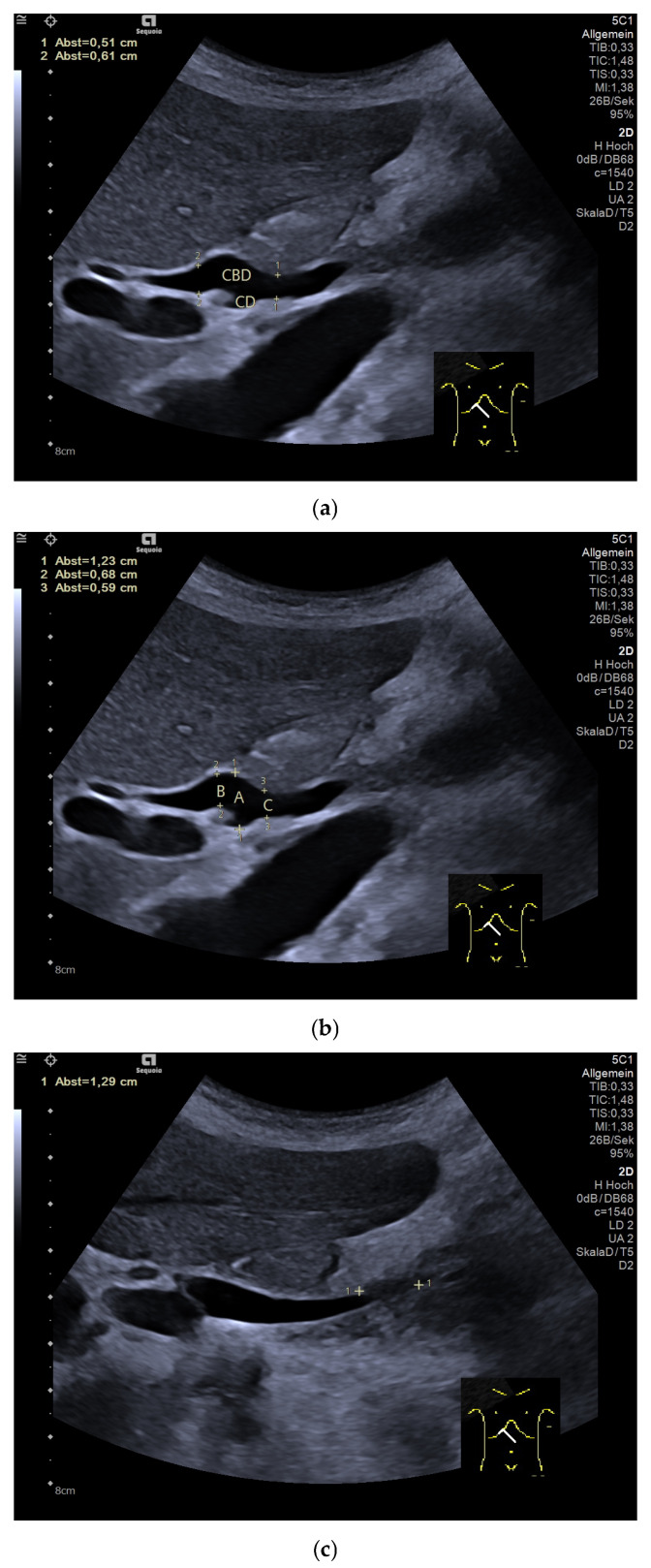

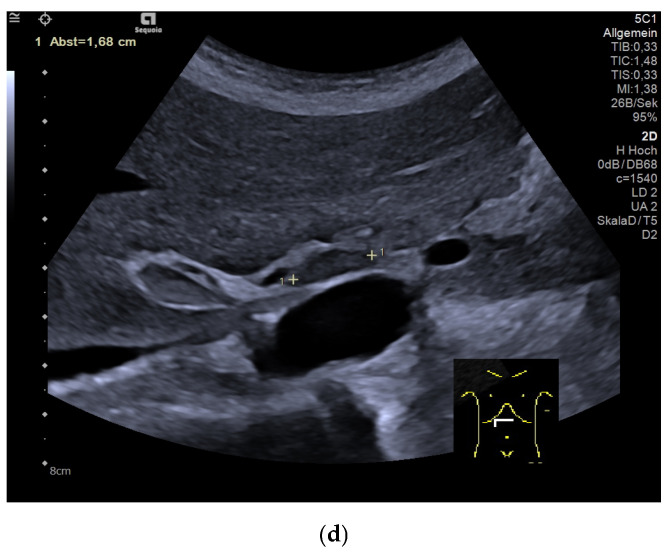
Normal common bile duct (CBD) with cystic duct (CD) orifice. (**a**) With two different explanations of measurements. CBD with different measurements between 6 mm and 12 mm depending on the specific measurement localization (**b**). Distal part of the CBD with the papilla in between the markers (**c**). Cystic duct lymph node next to the cystic duct orifice between portal vein and inferior vena cava (**d**).

Variable and sometimes deep inhalation and the left lateral position can be helpful for a better assessment of the bile ducts. The assessment of the distal common bile duct and papilla requires a certain amount of experience and manual dexterity. The proximal CHD may also be aligned with the intercostal region in the hepatic hilus via the main portal vein and the crossing proper hepatic artery as landmarks. The maximum anteroposterior diameter is measured from the inner-to-inner wall, perpendicular to the course of the duct in longitudinal view and sometimes with subcostal alignment with deep inhalation [21,27], identifying and avoiding the conjunction of the cystic duct with a distance of 10 mm to both sides in order to avoid overestimation [Figure 3b]. Ultrasound is sensitive in the detection of gallstones with a variable sensitivity of up to >90%, but the method of choice to detect stones in the common bile duct and microlithiasis is endoscopic ultrasound [28,29,30]. The use of Color Doppler Imaging (CDI) methods is additionally valuable in the differentiation of anatomic structures and avoids the misinterpretation of B-mode US [31]. For mobile patients, better visualization can sometimes be achieved in a standing position. Examination technique videos that illustrate the measurement technique have been published [32] [Figure 3].

### 4.2. Method and Technique of Measurements

#### 4.2.1. Extrahepatic Bile Duct (EHD)

- Diameter measurements are conducted at the maximum anteroposterior diameter from the inner-to-inner wall, perpendicular to the course of the duct in longitudinal views and variable deep inhalation at the porta hepatis (proximal), at its most distal aspect close to the head of the pancreas (distal), and midway between these points (middle) (Table 1). Due to the oval cross-section of the bile duct, the anteroposterior diameter is often smaller than the transverse diameter.
- The wall thickness of EHD is measured at the mid-duct as the distance between the inner and outer edge of the internal hypoechoic layer. When the wall thickness is asymmetric, measurements are obtained at the thickest points ([33,34] for EUS). Additionally, the ratio of EHD wall thickness to diameter can be estimated [35].

#### 4.2.2. Intrahepatic Bile Ducts (IHBDs)

**Figure 4:**
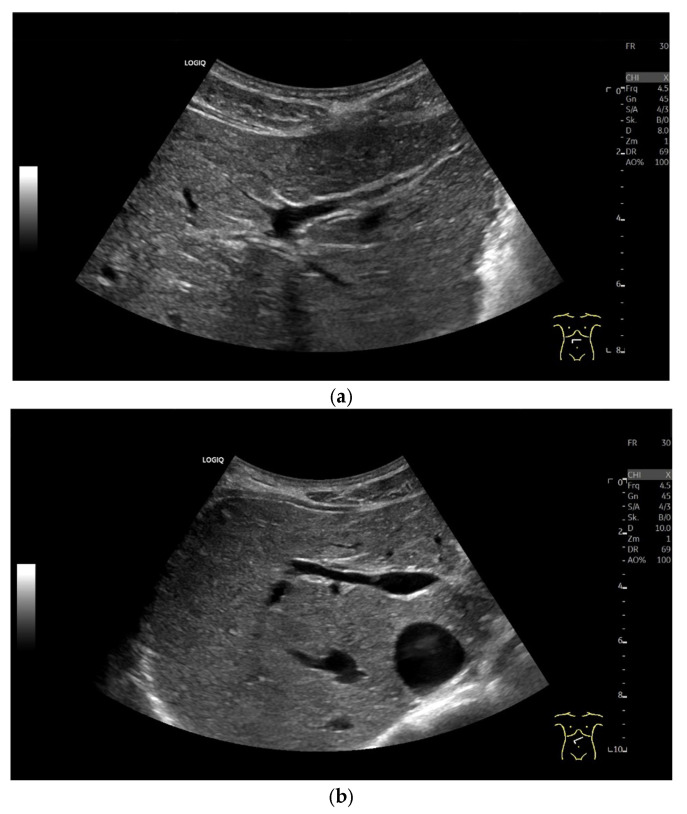
Intrahepatic bile ducts. Non-dilated, non-demarcated bile ducts of the left (**a**) and right liver lobe (**b**).

The central intrahepatic bile ducts should be visualized through a sagittal to right oblique (shoulder–navel section) and sometimes transverse subcostal alignment of the transducer [2,21] (Table 1). An intercostal approach is rarely required. Frequently, the intrahepatic bile ducts are only visible when dilated. The right and the hepatic ducts, as well as the segmental branches, usually run parallel and ventral to the respective portal vein branches [Figure 4]. We recommend measuring the maximal anterior–posterior diameter at the level of the portal bifurcation from inner-to-inner wall [21]. A comparison with the adjacent portal vein diameter is not recommended.

In liver cirrhosis, the arterialization of the liver is increased as compensation. In the left lobe of the liver, the prominent segmental hepatic arteries run parallel to the portal branches and should not be misinterpreted as dilated segmental bile duct branches. This differentiation is easily possible using Color Doppler Imaging.

## 5. Reference Values and Influencing Factors

### 5.1. EHD Diameter

The reference values for the diameter of the EHD given in the literature show large variations: 5–9 mm [3], <7 mm [23], and <7–8 mm (up to 10 mm) [12]. An overview of the confirmed and discussed influencing factors is given in Table 2.

#### 5.1.1. Age as an Influencing Factor

Some studies report a correlation between EHD diameter and age (<50 years: 3.2 ± 0.9 mm, >50 years 4.2 ± 1.2 mm [27]), or particular upper limits for the elderly: <7.9 mm [36] and <8.5 mm [27], while others reported normal values of up to 10 mm in elderly or old cohorts [12,23,24,27,37,38,39]. Some try to define a CBD diameter increase of ~0.2 mm per age decade and an increased EHD diameter of 1 mm in post-cholecystectomy subjects compared to the healthy population [3,23].

#### 5.1.2. History of Cholecystectomy as an Influencing Factor

Due to several other influencing factors, no consensus has been found by Sienz et al. regarding the impact of cholecystectomy on the EHD diameter before 2010 [3]. Different reference values have been stated for patients after a cholecystectomy of <10 mm [12,23,24], and with a pathologic cut-off of >14 mm after cholecystectomy [12,23].

Kratzer et al. observed a EHD diameter of >7 mm in 45% of patients with a history of cholecystectomy compared to 18% of patients without this history [23]; this was without statistical significance in a large cohort of 8534 patients. On the other hand, the positive correlation between age and EHD diameter could be confirmed.

A longitudinal study assessing EHD before and after cholecystectomy found a slight increase in EHD diameter 3 and 6 months postoperatively, but no increase within 24 h of surgery. The increase was significant, yet small and still below the pathological threshold of 7 mm [25].

Kaim et al. studied the width of the common bile duct in asymptomatic patients over 75 years old. Other criteria were the presence or absence of cholecystolithiasis and a condition after cholecystectomy. The mean width (±SD) of the EHD was 6.5 ± 2.5 mm. Patients without cholecystolithiasis (6.2 ± 2.3 mm) and patients after cholecystectomy (8.7 ± 2.9 mm) were statistically significantly different (*p* < 0.0001). Cholecystolithiasis (6.0 ± 1.6 mm) was not associated with EHD enlargement [12]. In a subgroup of patients older than 92 years of age, normal values of up to 14 mm were observed.

#### 5.1.3. History of (Bariatric) Surgery

An American retrospective study reassessed EHD diameter before and around up to 2 years after bariatric surgery [40]. By evaluating data from 269 patients, they found an increase in EHD diameter of 1.4 mm (95% CI 0.096, 0.18) following Roux-en-Y gastric bypass (RYGB), independent from prior cholecystectomy in the RYGB group. EHD diameter following sleeve gastrectomy (SG) showed smaller changes (0.5 mm, 95% CI 0.007, 0.11). But for patients without a prior cholecystectomy within the SG cohort, a significant increase in EHD diameter of 0.8 mm (95% CI 0.02, 0.14) post surgery could be stated. The cause of this phenomenon is unclear [40]. Roux-en-Y gastric bypass is a difficult situation. Obesity per magna and the altered anatomy after gastric bypass make the diagnosis of choledocholithiasis more difficult using US. As a result of the altered anatomy, the EHD is no longer completely visible in the EUS. After gastric bypass, the papilla can no longer be reached from retrograde using the conventional ERCP technique. Complex therapeutic techniques are required, the application of which demands a reliable diagnosis.

#### 5.1.4. Sex as a Possible Influencing Factor

While most studies failed to demonstrate a significant impact of sex on EHD diameter [41,42,43], Kratzer et al. showed a 1.4–1.5-fold higher chance of a EHD diameter >7 mm in females [23], and a slight association between gender and duct dilatation was demonstrated by Matcuk et al. [21].

#### 5.1.5. Ethnicity as a Possible Influencing Factor

The same applies in terms of ethnicity, despite well-known cultural differences in diet and the prevalence of gallstones. Lower average and upper limit values 3.6 ± 0.1 mm (1.8–5.9) were measured in Ethiopians compared to previously conducted studies [39], but no difference between different ethnicities was stated by Matcuk et al. [21].

#### 5.1.6. Physiological Factors and Comorbidities

The EHD diameter was found to increase with inhalation [44]. Due to the oval cross-section of the bile duct, the anteroposterior diameter is often smaller than the transverse diameter. Dynamic changes during an episode of bile colic could be documented with ultrasound and CT in a case study by Jafari et al. [45]. They measured EHD diameters within 72 h after admission, and values of 17 mm on admission were documented with confirmed choledocholithiasis, 4 mm, and again 14 mm versus 4 mm during bile stone passage. Therefore, the natural course of a possible underlying stone passage should always be considered when comparing different measurements at different times.

In the postprandial state, a decrease of up to 2 mm within 20 min was described, while other studies did not show a difference in EHD diameter between fasting and non-fasting subjects, as reviewed by Sienz et al. [3].

The influence of weight and body mass index remains controversial [46].

Furthermore, an increase in diameter is stated with periampullary diverticula [47], autosomal dominant polycystic kidney disease [48], and graft-versus-host disease [49].

#### 5.1.7. Drugs as Influencing Factors

An Iranian study investigated the influence of opium consumption on EHD diameter [26] in 1599 individuals. They found a statistically, but merely clinically significant increase in the diameter, especially in heroin consumers (5.54 ± 1.95 versus 4.74 ± 1.34 mm). An increase in EHD diameter for opium-addicted persons and patients on methadone was confirmed in other studies [50,51,52,53].

Other drugs have been suspected to affect biliary tract motility indirectly. For tegaserod, a prokinetic drug used for the treatment of irritable bowel syndrome with constipation, an American placebo-controlled study with healthy female subjects did not show an effect on EHD diameter both in fasting and postprandial states [54]. Ursodeoxycholic acid (UDC) exerts significant inhibitory effects on interdigestive gallbladder contractility, but its possible effects on CBD diameter have yet not been investigated [55].

Commonly used drugs for anesthesia like fentanyl and sufentanyl had no effect on the EHD diameter, as shown in a double-blind, placebo-controlled study [56].

#### 5.1.8. Influence of Imaging Methods on Measured EHD Diameter

Due to different spatial resolutions and dependencies on patient’s characteristics, EHD are visible differently in ultrasound compared to other imaging methods (Table 3). This may result in various measurement results. Previous studies reported discordant values for EHD diameter between ultrasound and following ERC with limited clinical value for direct comparison [57,58]. In general, there is agreement between ERC and ultrasound in diagnosing duct dilatation (specificity 90%). However, there was significant disagreement between the two techniques in the detection of non-dilatation, with dilated or ‘dilatable’ systems being missed by ultrasound in 52% of cases (11 out of 21) in which ERC found them [57].

The main reason for incongruency is measurement in different, not standardized regions. For example, during transabdominal ultrasound investigation, the junction of cystic and hepatic ducts is not always definable. In general, the radiographic magnification and sonographic underestimation of imaging may play a role. In the case of prior cholecystectomy, the loss of bile duct wall elasticity with reduced capacity for rapid caliber change after contrast injection is discussed [57].

A recent study comparing CT and ultrasound for acute cholecystitis found excellent agreement for bile duct diameter (ICC = 0.848) [59]. Differences between bile duct diameters were small (nearly all < 1 mm). Similar results were found in a direct comparison between MRI and ultrasound [11].

### 5.2. EHD Wall Thickness

Only a few studies deal with the reference values for the EHD wall thickness, mostly investigated using intraductal or endosonographic ultrasound [33,35] (see Table 4).

#### Influencing Factors on EHD Wall Thickness

Apart from in malignant processes, increased wall thickness has been described in inflammatory processes, such as cholangitis, primary sclerosing cholangitis (PSC), IgG4 cholangiopathy, or earlier biliary catheter placement [35,63]. On the other hand, uncomplicated inflammatory bowel disease or choledocholithiasis had no influence [33].

### 5.3. Diameters of Intrahepatic Bile Ducts (IHBDs)

Sonographic studies evaluating IHBDs are scarce and if assessed, normal values are not established. In previous studies, intrahepatic bile ducts were judged as normal if the segmental or more peripheral ducts could not be delineated [3,12].

With improving ultrasound techniques in recent years, the diameters of second-order bile duct branches <2 mm were considered normal [24].

In the cohort of Matcuk et al. [21], the intrahepatic ducts were assessed at the level of the portal bifurcation via 1484 normal ultrasound examinations. The mean normal values for RIHD and LIHD were 1.9 ± 1.9 mm and 1.9 ± 1.1 mm.

An MRCP study analyzing the whole biliary tree at 14 measurement points defined an upper normal limit of 2 mm for intrahepatic bile ducts [64]. According to Venkatesh et al., intrahepatic bile ducts are usually seen in standard MRCP examinations in non-dilated ducts till the segmental ducts (third order). If many fourth- or even fifth-order ducts can be depicted, disease and downstream strictures should be excluded [65]. In another MRCP study, the authors concluded that the intrahepatic ducts should not exceed a diameter of 3 mm [66].

#### Influencing Factors on IHBD Diameters: Imaging Method and History of Cholecystectomy

Similarly to EHD diameter, age and history of cholecystectomy could be relevant influencing factors. Only a few of the sonographic studies evaluating EHD diameter assessed the dilatation of IHBDs as well. In the cohort of McArthur et al. [24], only 0.4 to 4.0% of the 893 patients (ages 17–100 years) exhibited dilatation of the central IHBDs. The highest frequency was seen in the group with a history of cholecystectomy more than 10 years ago (5 of 117 patients), with 4.0% [24]. In this study, history of cholecystectomy was the main influencing factor, with only a mild, non-significant increase with age. There is no known correlation between age and the dilatation of IHBDs.

Even with CT and its lower spatial resolution, mild dilatation could be documented in 31.2% (24 of 77) of EHD and only in 18.2% (14/77) of IHBDs in asymptomatic individuals after cholecystectomy compared to 2.6% in the control group [67]. IHBD dilatation was defined as ducts being visualized outside the porta hepatis.

Compared to these asymptomatic subjects, the frequency of IHBD dilatation was about 56% in a study evaluating CT in symptomatic post-cholecystectomy patients (n = 118) [68].

According to the authors’ experiences, the sonographic demarcation of IHBDs is more often seen in the left than in the right liver lobe. To our knowledge, there are no sonographic studies evaluating this impression. A prospective study investigating changes post cholecystectomy with MRCP confirmed the dilatation of IHBDs 3 and 6 months post surgery in 48 patients [64]. They were measured at three different intrahepatic points in each hepatic lobe (near bifurcation and the widest segmental ducts). The widest diameters in the left hepatic lobe were seen most frequently in Segment 3 (26 of 48 patients) and in the right hepatic lobe in Segment 5 (21 of 48 patients). Although statistically significant, the changes remained small and below the upper limit of 2 mm: for the right IHBD, from 1.00 (0.90–1.20) mm to 1.70 (1.20–1.90) mm, and for the left IHBD, from 1.00 (0.95–1.15) mm to 1.68 (1.25–2.11) mm before and 6 months after cholecystectomy. The values for the left and right hepatic ducts differed slightly according to the measuring direction in MRCP (anteroposterior versus latero-lateral) (between 3.2 and 3.6 before and 3.4–4.0 mm post surgery). Possible explanations for the most frequent IHBD dilatation, at least in Segment 3, include (a) the suboptimal adjustment of the IHBD drainage system after cholecystectomy, with lower drainage capacity of the left lobe compared to the right lobe, and (b) increased pressure in the IHBD of Segment 3 (and 5) due to the necessity for bile transport against gravity.

Considering the different possible explanations for EHD or IHBD dilatation, clinical and laboratory findings—especially elevated bilirubin levels [68]—are the main arguments for further diagnostics or intervention (see Table 5).

## 6. Reference Values and Documentation

EHD diameter: <7 mm, but always considering influencing factors mentioned above.

EHD wall: pathologic cut-off: >1.5 mm (compare Table 4).

EHD wall/diameter ratio: normal about 30%, in PSC about 59% [35].

Intrahepatic bile ducts: <2–3 mm [21] and <2 mm [2,24,64]. Pathologic cut-off: >3 mm.

Cystic duct: Reference values are not yet established.

Papilla: Reference values are not yet established.

## 7. Practical Tips, Tricks, and Recommendations

Each sonographic finding must be interpreted in the context of clinical and paraclinical settings. The estimated probability of a relevant bile pathology/obstruction of outflow can be divided into the following categories:

- Low: unremarkable liver and cholestasis parameters and unspecific complaints;
- Moderate: unspecific right-sided upper abdominal symptoms and laboratory parameters, which may also be related to exogenous noxious agents and/or steatosis hepatis;
- High: typical right-sided upper abdominal pain, typical elevated liver and cholestasis parameters.

For a detailed definition of pretest probability, see (among others) the guidelines for prophylaxis, diagnosis, the treatment of gallstones [69], and bile duct strictures [34]. The above-mentioned influencing factors (e.g., age, post cholecystectomy, known cholecystolithiasis, and postprandial state) (see Table 2) should be recalled. A comparison with earlier US examinations might be helpful if possible. This is one of the reasons for exact documentation. Table 6 summarizes the diagnostic algorithms for findings on common bile ducts (dilated or non-dilated) depending on their transabdominal sonographic visibility.

### 7.1. How to Deal with Aerobilia and Biliodigestive Anastomosis

Biliodigestive anastomosis (BDA) as part of hepatobiliary pancreatic surgery can be performed as hepaticoduodenostomy or hepaticojejunostomy, the latter being the recommended procedure. It results in the loss of sphincter of Oddi with the consecutive possible ascension of bacteria into the bile system and reflux of air. The standard anastomosis is carried out proximal to the cystic duct (together with cholecystectomy if not earlier performed) and 2–3 cm below the hepatic duct bifurcation [71]. To our knowledge, there are no studies concerning sonographic anastomosis measurements. An estimation can be made indirectly via the common usage of 1 to 4 stents from 8 to 14 Fr (3–5 mm) for biliary drainage therapy [72]. Another study comparing different hepaticojejunostomy techniques on 267 patients reported anastomotic diameters of about 1.5 cm (0.7–3.0 cm) [71]. Savic et al. evaluated choledochoduodenal anastomosis function in 50 Serbian patients following operation for benign biliary obstruction [73]. They found a decreasing diameter from day 7 to day 30 post operation, from 14.59 ± 4.13 mm (min. 10–max. 25 mm) to 7.73 ± 1.40 mm (mind. 6-max. 11 mm)—the postoperative values being statistically lower than the preoperative values.

Aerobilia following biliary interventions considerably disturbs the measurement of bile duct diameters due to artifacts. Sometimes, the relocation of air bubbles into other parts of the bile ducts can be achieved by changing the position of the patient, and may result in a better view for measurements. In the cohort of Savic et al., aerobilia was present in 91.7% of patients during the first postoperative week, but decreased towards 16.7% on day 30 [73]. Aerobilia was reported in 42% of the 47 cases after endoscopic sphincterotomy and in 50% of patients after surgical sphincterotomy, and decreased to 11% in the cases 25 years after the intervention [74].

### 7.2. Variations

#### Congenital Bile Duct Malformations

Congenital bile duct malformations are associated with ductal dilatation and cystic ectasia (Table 7). They lead to increased gallstone formation, cholangitis, and strictures. Examples are the choledochal system classified according to Todani or bile duct duplication [75,76]. Different variants of bile duct orifices and accessory ducts must be distinguished. They are usually detected in duct imaging procedures such as MRCP or ERCP, or diagnosed intraoperatively. A severe malformation is biliary atresia, with biliary obstruction in the first days of life. These children need a hepatoportoenterostomy procedure (Kasai operation) or require a liver transplantation.

## 8. Controversies

### 8.1. Should We Always Image the Cystic Duct During Measurements of the Extrahepatic Bile Duct?

In an early ultrasound study published in 1989, the cystic duct could be visualized in 51% of patients with a normal common bile ducts and normal gallbladders [82]. In 86% of the participants, the cystic duct joined the distal part of the common bile duct, and in 14%, the middle part. In 95% of the patients, the cystic duct entered the CHD at its dorsal side, in 5%, ventrally. The average diameter was found to be 1.8 mm. The length of the cystic duct was 1–23 mm, with an average of 9.8 mm [82]. In the case of dilatation of the cystic duct, a tortuous course in the liver hilus was seen in 80% and a tubular course in 20% [83]. The tortuous pattern should not be confused with cavernous vessels. The tubular pattern was dorsal and parallel to the common bile duct and resembled a duplication of the common bile duct. More recent transabdominal ultrasound studies on the visualization of the cystic duct are not available. The diameter of the common bile duct depends to a small extent on the section where the measurement is taken [84]. It is slightly wider distally—i.e., after the confluence of the cystic duct—than in the middle and proximal parts [27].

From the authors’ experience, the visualization of the cystic duct depends on the quality of sonographic imaging in the individual patient, the distance between the bile duct and the body surface, the upstream artifacts in the gastric antrum and duodenum, and the diameter of the distal common bile duct. If the common bile duct is large, it is easier to visualize the junction of the cystic duct.

### 8.2. Is It Necessary to Measure the Extrahepatic Bile Duct in All Parts?

In the case of common bile duct obstruction, the assessment of the entire common bile duct is necessary to determine the level and cause of blockage. The Extrahepatic bile duct is not equally wide in all parts. Bachar et al. determined the mean lumen width of the Extrahepatic bile duct in the proximal, middle, and distal sections [27]. However, there is no information on how often all parts were visible. The visualization of the EHD, in general, is reported for at least 94% of examinations [19]. In the authors’ experience, it can be particularly difficult to assess the prepapillary region when the duct is not dilated.

Alternatively, the bile duct can be assessed using endoscopic ultrasound (EUS) and MRCP. The bile duct can be adjusted using both radial and longitudinal EUS transducers. The biliary system is assessed using the longitudinal probe in five positions in the post bulbar duodenum, duodenal bulb, and stomach [85]. The accessibility of the longitudinal system versus radial system from the middle to distal bile duct and from the proximal bile duct to the hepatic portal region was 90.1%/78.2% versus 90.9%/7.1%, respectively [86]. In the middle to proximal part of the extrahepatic bile duct, the longitudinal transducer was significantly superior. All the other data were not significantly different. In the authors’ own experience, the extrahepatic bile duct is slightly compressed during the coupling of the EUS transducer to the wall of the post bulbar duodenum and duodenal bulb. It is then sometimes not comprehensible that the duct is measured as dilated when using other imaging methods. This compression effect must be considered. If the proximal part of the extrahepatic bile duct is assessed via the stomach, it is far away from the transducer and this compression effect does not exist.

### 8.3. How Often Can the Papilla Be Visualized?

The distal section of the common bile duct is visualized dorsally to the head of the pancreas and traced up to the duodenal structures. Before it joins the papilla, the tubular duct narrows slightly and tapers. This corresponds to the passage through the circular sphincter. In the authors’ experience, the visualization of the papillary region depends on the depth of penetration and quality of visualization. To the best of our knowledge as authors, we are not aware of any ultrasound studies on how often the papilla can be visualized using transabdominal ultrasound. The standard for the assessment of the papilla of Vater is duodenoscopy using a sideview endoscope. Endosonographically, the experienced examiner should always be able to visualize the papilla of Vater tracking to the mouth of the common bile duct and pancreatic duct, provided that the passage into the post-bulbar duodenum is not anatomically obstructed. This applies to longitudinal and radial probe systems. The papilla of Vater was visible in 82.2% versus 85.9% of cases, when comparing the longitudinal transducer with the radial transducer [86]. The reference values are not yet established. Our own non-published data found mean values of 13 × 7 mm using the endoscopic ultrasound approach (100 patients).

## 9. Future Perspectives

Data from large registries will help to clarify the role of bile duct measurements in many diseases and conditions. Artificial intelligence and deep-learning and neural-network methods are applied in many medical fields and in the analysis of sonographic imaging. Measurements of volume and wall thickening might be automated. However, its translation into clinical practice has still to overcome many hurdles and requires prospective clinical evaluations.

## 10. Conclusions

Ultrasound examination determines the diameter of the extra- and intrahepatic bile ducts and the level of outflow obstruction. The assessment of the prepapillary part of the common bile duct can be more difficult. The largest diameter of the extrahepatic bile duct should be measured in the case of right-sided upper abdominal complaints, abnormal liver and cholestasis laboratory parameters, and if the extrahepatic bile duct appears visually enlarged. Since the diameter of the extrahepatic bile duct is age-dependent and may be larger after cholecystectomy or bariatric surgery, the findings must always be viewed in conjunction with the clinical presentation and laboratory values. In asymptomatic patients with a history of cholecystectomy, normal laboratory parameters, and a dilated bile duct without signs of obstruction, no further diagnostic workup is required.

If the cause of obstruction cannot be confirmed and a diagnosis cannot be established in symptomatic patients with a dilated extrahepatic bile duct or increased cholestasis parameters, endosonography or MRCP should be performed [69,87,88]. Papillary adenomas are excluded using side-view duodenoscopy or longitudinal EUS during the examination.

## Figures and Tables

**Table 1:** What you should practice when learning how to carry out ultrasound of bile ducts.

Anatomical Structure	What Should I Do?
Extrahepatic bile ducts	Complete visualization of the bile duct from the hepatic bifurcation to the junction with the duodenum. Locate distal CBD in transverse section within the pancreatic head (mimicking a pancreatic cyst due to transversal plane). Turn into the longitudinal course of the CBD by rotating into the right oblique (shoulder–navel section) subcostal position and follow it to the liver hilus, and if possible distally to the duodenum. Visualization of the CHD at the liver hilus with intercostal alignment of the probe. Explanation: the shape of the duct is important, i.e., carry out complete, or only spindle-shaped dilatation over the whole length as normal variation, or due to the confluence of the cystic duct. Measure where the diameter is the largest from inner-to-inner wall.
Intrahepatic bile ducts	Look for the presence of segmental bile duct branches in the liver segments parallel to the portal branches and segmental arteries. If yes—is it segmental congestion or congestion affecting the whole liver? In case of liver cirrhosis—is it really the bile ducts or compensatory enhanced segmental arteries (Color Doppler Imaging)?

**Table 2:** Overview of influencing factors resulting in EHD dilatation. (++) strong evidence and proven correlation, (+) some evidence and plausible correlation, ((+)) little, partly divergent evidence, and (−) sufficient evidence to state no influence.

Factors Influencing Increase in EHD Diameter	Correlation/Evidence
Age	++
History of cholecystectomy	++
Inhalation	+
Periampullary diverticula	+
History of (bariatric) surgery	+
Autosomal dominant polycystic kidney disease	+
Graft-versus-host disease	+
Opioid or methadone treatment	+
Female sex	(+)
Anesthetic drugs (propofol, fentanyl, sufentanyl)	−
Ethnicity	−

**Table 3:** EHD diameter in different imaging modalities. TAUS = transabdominal ultrasound, US = ultrasound, MRI = magnetic resonance imaging, EUS = endoscopic ultrasound, and CT = computed tomography.

Imaging Modality	N (Female/Male)	Mean Age (SD)	EHD (mm)	Study Details	Reference
Laparoscopic US (LUS)	253 (167/86)	43.5	LUS: 3.7 mm TAUS: 4.0 mm	Obese patients, mean BMI 48, LUS vs. TAUS	Kothari et al. (2013) [46], USA
MRI	76 (35/41)	43.9	TAUS: 3.17 mm MR: n.s.: 3.14–3.30 mm depending on used sequence	Randomized controlled trial, small n.s. increase with age (0.0155 mm per year)	Prpic et al. (2007) [11], Croatia
ERC	50 (39/11)	58	TAUS all: 7.9 ± 3.3 mm ERC all: 13.1 ± 4.5 mm TAUS normal: 6.2 ± 2.5 mm ERC normal: 11.5 ± 4.0 mm (significant)	TAUS 2–3 h prior to ERC, all patients post CCE and symptomatic Normal subgroup n = 22 without biliary pathology	O’Connor et al. (1985) [57], GB
CT	353	59.7 (18.0)	Mean difference US vs. CT—0.27 mm for dilated EHD	Imaging for suspected acute cholecystitis	Schuster et al. (2023) [59], USA
MRCP/CT/EUS	109 (74/35)	71 (14.0)	10.3–11.0 mm, n.s.	EUS for work up of dilated EHD, retrospective, different clinics	Ferreira et al. (2024) [60], Portugal

**Table 4:** Wall thickness of EHD estimated using different ultrasound imaging methods. SD = standard deviation. PSC = primary sclerosing cholangitis.

N (Female/Male)	Mean Age (SD)	Imaging Modality	Wall (mm, SD)	Study Background	Reference
50 (29/21)	56.9 y (16.3)	Transduodenal EUS	Controls: 0.8 mm (0.4) PSC: 2.5 mm (0.8) Cut-off: >1.5 mm	British, PSC diagnostic	Mesenas et al. (2006) [35]
95 (n.a.)	63.3 y	Intraductal US	Controls: 0.6 mm (0.3)	Japanese, retrospective	Tamada et al. (1999) [33]
60 (29/31)	55 y	Radial EUS	Controls (cholestasis): 1.1 mm (0.5) Cut-off: >1.5 mm	Turkey, prospective, cholestasis vs. cholangitis	Alper et al. (2011) [61]
21 (13/8)	59 y (29–79)	Linear EUS	Controls: 0.39 ± 0.14 mm PSC: 0.89 ± 0.59 mm	Croatian, prospective, elastography for PSC	Rustemovic 2010 [62]

**Table 5:** Documentation with clinical relevance.

Indication	Anatomical Structure
Routine	Extrahepatic bile duct (EHD) with measurement of widest diameter and reporting which parts could be visualized (bifurcation, hilus, retro pancreatic)
With clinical question	Intrahepatic bile ducts Thickness of EHD wall

**Table 6:** What is concerning—red flags, caveat in algorithms depending on sonographic findings (based on [34,44,69,70]). W&W = watch and wait.

**(A): Non-dilated common bile duct: EHD < 7 mm.**			
**Transabdominally visible in all parts (bifurcation, hilus, retro pancreatic)**			
**Low** probability	Moderate probability		High probability
Stop biliary diagnostic	W&W possible Consider further diagnostics (MRCP or EUS depending on availability)		Further diagnostics indicated (EUS, MRCP)
**Transabdominally not visible in all parts (e.g., only in hilus)**			
**Low** probability	Moderate probability		High probability
W&W Look for other reasons	Further diagnostics indicated (MRCP or EUS depending on availability)		Further diagnostics indicated (EUS > MRCP)
**(B): Dilated extrahepatic bile duct (EHD): What is normal, what is concerning, and when to clarify further?**			
**What is normal?**	**What is concerning?**		
Dilated EHD **without symptoms/elevated cholestasis parameters**	Dilated EHD **with symptoms/elevated cholestasis parameters**		
No symptoms, no elevated liver or cholestasis parameters, no evidence of outflow obstruction in high quality US	Right-sided upper abdominal pain/colic ± increased liver and cholestasis parameters, other B-symptoms		
Known change without symptoms with normal laboratory and normal imaging	Cholecystolithiasis		
	Simultaneously dilated pancreatic duct > 2.5 mm		
	Pancreatic cystic lesions/IPMN		
	Solid lesion of the pancreatic head		
	Acute/chronic pancreatitis		
	Increase in dilatation of a presumed benign bile duct dilatation		
**EHD dilated (e.g., >7 mm)** **and transabdominally not visible in all parts (e.g., only in hilus)**			
**Low** probability	Moderate probability		High probability
Consider further diagnostics (MRCP, EUS, including endoscopic assessment of the major papilla with exclusion of a papillary adenoma)	Further diagnostics indicated (EUS, MRCP)		Further diagnostics/therapy indicated
**EHD dilated (e.g., >7 mm)** **and transabdominally visible in all parts (bifurcation, hilus, retro pancreatic), preserved respiratory variability, spindle-shaped)**			
**Low** probability	Moderate probability		High probability
Stop biliary diagnostic	W&W possible Consider further diagnostics (MRCP, or EUS depending on availability, including endoscopic assessment of the major papilla)		Further diagnostics indicated (EUS > MRCP)
**(C): Visible bile duct stones regardless of EHD diameter**			
**Visible extrahepatic stones regardless of EHD diameter**			
**With symptoms/elevated cholestasis parameters**		**Without symptoms/without elevated cholestasis parameters**	
Therapy indicated		Post cholecystectomy	Gallbladder in situ
		Therapy indicated	W&W (elder patients), consider therapy
**Intra** **hepatic stones regardless of EHD diameter**			
**With symptoms/elevated cholestasis parameters**	**Without symptoms/elevated cholestasis parameters**		
Therapy indicated	W&W recommended		

**Table 7:** Congenital changes in the bile ducts.

Congenital Changes in the Bile Ducts		
Nature of Changes	Description	Clinical Impact
Biliary atresia (BA) [77,78]	Obliterative (inflammatory) fibrosing cholangiopathy with obstruction of the intra- and extrahepatic bile ducts. *Variations*: Isolated BA; BA Splenic malformation; Cystic BA. *Types according to the level of obstruction:* Type 1: Atresia of the distal extrahepatic bile duct; Type 2: Atresia of the common bile duct at different levels, presence of a cyst at the hilum; Type 3: Obstruction of the entire extrahepatic biliary system and intrahepatic bile ducts at the hilum; gallbladder, cystic duct, and bile duct may be patent; Type 4: Complete obstruction of all extra- and intrahepatic bile ducts [78].	Jaundice in the first weeks of life. Association with other severe congenital anomalies. Type 1 and 2 are correctable. Treatment options: Kasai operation, liver transplantation [77].
Common bile duct duplication [79]	Presence of a septum within the CBD or an accessory CBD [79]. Not to be confused with the double ductal structure in deep-opening ductus cysticus.	Associated with pancreaticobiliary maljunction and congenital biliary dilatation in children [80].
Choledochal cysts (Congenital choledochal malformation) [75,76]	Cystic dilatation of the intra- and extrahepatic bile ducts. On ultrasound: Segmental bile duct dilatation, cystic lesions adjacent to the bile ducts. *Classification according to Todani* [75,76]: Type I: Cystic or fusiform dilatation of the common duct; right and left hepatic ducts and intrahepatic bile ducts are normal; Type II: Diverticulum of CBD; Type III: Cystic dilatation of distal common bile duct, intramural in the duodenal wall; Type IV: Multiple either intrahepatic or extrahepatic cysts, or exclusively extrahepatic bile ducts; Type V: Single or multiple intrahepatic cysts with normal extrahepatic bile ducts; Caroli disease.	Diagnosis via MRCP, ERCP. Increased prevalence of gallstone formation with subsequent cholangitis and biliary strictures. Increased risk of carcinoma [81].

## Data Availability

The data presented in this study are available upon request from the corresponding author. The data are not publicly accessible, as the personal rights of the patients involved must be respected.
